# Multi-Frequency Bioimpedance Analysis in Practice: A Review of Validated Prediction Equations for Key Body Composition Parameters

**DOI:** 10.33549/physiolres.935758

**Published:** 2025-12-01

**Authors:** Dávid KAMPO, Eva ZÁVODNÁ, Vlastimil VONDRA

**Affiliations:** 1 Institute of Scientific Instruments of the Czech Academy of Sciences, Brno, Czech Republic; 2 Department of Biomedical Engineering, Faculty of Electrical Engineering and Communication, Brno University of Technology, Brno, Czech Republic; 3 Department of Physiology and Pathophysiology, Faculty of Medicine, University of Ostrava, Ostrava, Czech Republic

**Keywords:** Bioelectrical Impedance Analysis, Body Composition, Prediction Equations, Total Body Water, Fat-Free Mass

## Abstract

This review provides a comprehensive synthesis of validated prediction equations for body composition assessment using single- and multi-frequency bioelectrical impedance analysis (BIA), covering studies published between 2000 and April 2025. While traditional models for estimating compartments such as total body water (TBW) and fat-free mass (FFM) have long been established, they often fail to reflect current populations and technologies. The review includes 43 studies that developed 98 unique equations for TBW, FFM, extracellular water (ECW), intracellular water (ICW), body cell mass (BCM), and bone mineral content (BMC), derived using reference methods such as deuterium dilution, DXA, or multi-component models. Most equations targeted FFM and TBW, with a noticeable lack of models for ECW, ICW, and BMC. The review identifies a geographic and demographic imbalance in study populations and emphasizes the need for updated, population-specific models. It also highlights the growing use of multi-frequency BIA devices to improve estimation accuracy. The findings support the continued refinement of BIA-based prediction models for broader clinical applicability and underscore the importance of external validation across diverse populations and health conditions.

## Introduction

The assessment of body composition has become increasingly important in clinical practice, nutritional research, and public health. Traditional anthropometric measures, while valuable, provide only limited information about body compartments. In contrast, bioelectrical impedance analysis (BIA) offers a non-invasive, simple, portable, and relatively inexpensive method to estimate body composition parameters, including total body water (TBW), extracellular water (ECW), intracellular water (ICW), fat-free mass (FFM), body cell mass (BCM), and body mineral content (BMC) [1–3].

BIA operates on the principle that biological tissues exhibit different conductive respectively resistive properties depending on their water and electrolyte content. Early applications of BIA primarily utilized single-frequency measurements; however, the development of multi-frequency BIA has enhanced the ability to differentiate between ECW and ICW compartments [2,4,5]. Unlike single-frequency models, which provide an overall estimate of total body water, multi-frequency BIA enables frequency-dependent analysis of impedance, thereby distinguishing extracellular and intracellular fractions – a capability essential in subjects with altered hydration. Although the raw impedance values can be measured directly, prediction of specific body compart-ments requires the application of validated regression equations derived against reference methods such as isotope dilution, dual-energy X-ray absorptiometry (DXA), or multi-compartment models [6,7].

The history of BIA research spans several decades, with seminal equations developed during the 1980s and 1990s by pioneers such as Kushner, Lukaski, Deurenberg, and others [8–10]. These early studies established the foundation for the clinical application of BIA, but many of the commonly used prediction models today still derive from these older datasets. Numerous reviews have summarized these historical equations; however, there is a growing recognition that these models may no longer adequately represent current populations, given shifts in demographics, lifestyles, and advances in technology [7,9–11].

Despite the publication of several review articles in recent years, a significant limitation persists: most analyses predominantly focus on prediction equations developed more than 20 years ago, often neglecting the substantial number of new studies published since 2000 [12,13]. Moreover, early studies frequently included relatively homogeneous samples in terms of ethnicity and age, whereas more recent research has embraced broader and more diverse populations [14,15].

The present review addresses this gap by systematically evaluating validated prediction equations for body composition assessment using multi-frequency BIA, focusing exclusively on studies published between January 2000 and April 2025. By doing so, it aims to offer an updated synthesis of contemporary models estimating TBW, ECW, ICW, FFM, BCM, and BMC. In addition, this review aims to provide a comprehensive resource for future studies validating and applying contemporary BIA prediction equations, thereby supporting further development and refinement of bioimpedance analysis methodology in clinical and research settings.

## Methods

A systematic literature search was performed to identify original studies that developed new prediction equations based on bioelectrical impedance analysis for estimating body composition compartments, specifically total body water (TBW), fat-free mass (FFM), extracellular water (ECW), intracellular water (ICW), body cell mass (BCM), and bone mineral content (BMC). The search was conducted in two major electronic databases, PubMed and Scopus, covering articles published between January 2000 and April 2025.

The search strategy combined terms related to bioelectrical impedance analysis and the development of prediction models for body composition estimation. Searches were adapted for each specific target compartment, including total body water, fat-free mass, extracellular water, intracellular water, body cell mass, and bone mineral content. Typical search strategies incorporated combinations of terms for BIA (“bioelectrical impedance” OR “bioimpedance” OR “BIA”) with terms related to model development (“prediction equation” OR “predictive model” OR “regression model” OR “estimation formula”) and the specific body compartment (“total body water” OR “fat-free mass” OR “extracellular water” OR “intracellular water” OR “body cell mass” OR “bone mineral content”), alongside terms indicating derivation processes (develop*, derive*, create*, formulate*). Filters were applied to restrict the search to articles published in English, involving human subjects, and to exclude studies focusing on bioimpedance spectroscopy (BIS).

In bioelectrical impedance analysis, body composition compartments are estimated using regression-based prediction equations that incorporate measured impedance values together with anthropometric and demographic variables such as height, sex, weight, and age. The formulation and validation of such equations constitute the main focus of this review. In contrast, bioimpedance spectroscopy applies mathematical modelling and mixture equations, such as the Hanai and Cole-Cole models, to resistance data measured across a broad frequency spectrum. This modelling approach enables the extrapolation of extracellular and intracellular fluid resistances from impedance values over a wide range of frequencies. Because BIS relies on biophysical modelling rather than empirically derived regression equations, and employs a substantially greater number of frequencies than conventional BIA, it represents a distinct analytical technique. Therefore, studies employing BIS were excluded from this review [92,93].

A total of 707 records were initially identified (251 from PubMed and 456 from Scopus). After the removal of duplicates, 298 unique records remained. These records were then screened in a two-step process. In the first step, titles and abstracts were reviewed for eligibility. Articles were excluded if they used BIA solely as a measurement tool without developing new predictive equations, if they only mentioned prediction without presenting a derivation of a model, if they utilized bioimpedance spectroscopy instead of conventional BIA, or if they focused exclusively on validating or applying existing models. In the second step, the full texts of the remaining articles were retrieved and reviewed in detail. Studies were excluded if they failed to report the development of new equations, if they lacked methodological details such as a description of the reference method or characteristics of the study sample, or if the statistical procedures used for model construction were inadequately described.

**Fig. 1:**
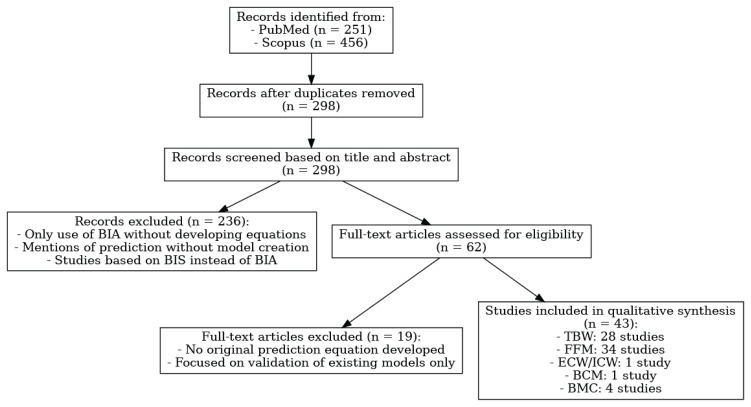
Flowchart of the study selection based on the records identified from PubMed and Scopus, screening, eligibility assessment, and final inclusion.

Following this selection process, 43 studies were included in the qualitative synthesis. Among them, 28 studies developed prediction equations for TBW, 34 for FFM, one for ECW/ICW, one for BCM, and four for BMC. The selection process is summarized in Figure 1.

From each study, the following information was extracted and organized for further analysis: the body composition compartment estimated, the authors and year of publication, the number of participants and their sex distribution and age range, the nationality and health status of the study population, the BIA device and measurement conditions used, the reference method applied for model validation, the developed prediction equations, and statistical measures of model performance, including the coefficient of determination (R^2^) and the standard error of estimate (SEE). These data formed the basis for the subsequent sections of this review.

## Description of reference methods in BIA

Accurate estimation of body composition compartments using bioelectrical impedance analysis requires validation against reliable reference methods. Various techniques have been employed across studies to establish prediction equations, each offering distinct advantages and limitations. This section provides an overview of the principal reference methods used in the development of BIA prediction models.

## Deuterium dilution technique

The deuterium dilution technique is one of the most commonly used reference methods for validating body composition estimates obtained by BIA. It is based on the administration of a known dose of deuterium oxide (^2^H_2_O) and the subsequent measurement of its equilibrium concentration in body fluids, allowing precise determination of total body water [16,17]. This method is considered highly accurate and has been widely employed in both paediatric and adult populations due to its ability to provide direct measurements of TBW inde-pendent of assumptions related to body tissue conductivity [18,19].

Several studies included in this review utilized deuterium dilution as the reference standard for developing prediction equations for TBW and FFM. For example, Lewis *et al*. [17] applied deuterium dilution to validate new BIA models in a population of Ugandan children, while Bedogni *et al*. [80] used it in a sample of healthy Italian adults to establish eight-point tactile electrode BIA prediction equations. In some studies, deuterium dilution has also been combined with sodium bromide dilution to separately determine extracellular water (ECW), since bromide ions remain restricted to the extracellular space [2]. The method typically involves collection of saliva, urine, or blood samples after an equilibration period, followed by analysis using mass spectrometry or infrared spectroscopy [16,22].

Despite its precision, the deuterium dilution method is relatively costly, requires specialized analytical equipment, and necessitates strict compliance with standardized protocols to minimize errors related to dose ingestion, equilibration, and sample analysis [17,23]. Nevertheless, its role as a gold standard for TBW assessment in validation studies remains uncontested, and it has been fundamental in calibrating BIA-based models across diverse age groups and ethnicities [18,20,24].

## Dual energy X-ray Absorptiometry (DXA)

Dual-energy X-ray absorptiometry has been extensively used as a reference method for body composition assessment, particularly for estimating fat-free mass and bone mineral content. DXA operates by passing two low-dose X-ray beams at different energy levels through the body, allowing differentiation between bone tissue, lean soft tissue, and fat mass [25,26]. It provides precise, region-specific, and whole-body measurements, making it a valuable tool for the development and validation of BIA prediction equations [27].

In the studies analysed, DXA served as a key reference technique for deriving new BIA-based models. For example, Sun *et al*. [28] employed DXA measu-rements in a large cohort of American adults to create prediction equations for TBW and FFM, while Bedogni *et al*. [21] combined DXA with deuterium dilution to validate body composition estimates in healthy populations. Moon [29] also highlighted the use of DXA as a gold standard for the assessment of body composition in athletic individuals.

Although DXA is regarded as highly accurate, it has certain limitations. The method is sensitive to variations in hydration status, technical calibration, and body size, particularly in obese individuals [30]. Moreover, despite the low radiation exposure, its application is restricted in specific groups, such as pregnant women, and requires expensive equipment and trained personnel [26,31]. Nevertheless, DXA remains one of the most widely accepted reference methods and continues to play a crucial role in the validation of BIA prediction equations across diverse demographic groups [25,28,30].

## Multi-component models

Multi-component models are considered among the most accurate reference methods for body composition assessment, as they integrate multiple independent measurements to partition the body into distinct compartments. Typically, these models combine data from several techniques, such as body density (hydrodensitometry), total body water (deuterium dilution), bone mineral content (DXA), and body weight, to construct three- or four-compartment models [32,33].

In the reviewed studies, multi-component models were utilized primarily to enhance the validity of BIA-derived prediction equations by minimizing assumptions associated with simpler two-compartment models (fat mass vs. fat-free mass). For instance, Sun *et al*. [5] applied a four-component model, in which body density, total body water, and bone mineral content were measured and the remaining component was calculated, to derive more precise TBW and FFM estimates through BIA. Their approach aimed to address the known limitations of single-method reference standards, such as hydration variability or bone mass underestimation [28,34].

The major advantage of multi-component models lies in their ability to correct for individual variability in tissue composition, such as differences in bone mineral density or hydration status [33,35]. However, their application is logistically demanding, requiring access to multiple measurement systems, specialized equipment, and standardized protocols to ensure consistency across methods [32,35]. Despite these challenges, multi-component models are often regarded as the gold standard for the validation of body composition assessment techniques and provide highly robust benchmarks for BIA-based equation development [28,34,35].

## Other reference methods

While deuterium dilution, DXA, and multi-component models represent the most frequently utilized reference methods in the reviewed studies, some prediction equations were developed using alternative validation techniques. The inclusion of these methods reflects the diversity of approaches adopted by researchers and underscores the importance of understanding the characteristics and limitations associated with each technique.

Hydrodensitometry, or underwater weighing, was employed in several studies to estimate body density, which subsequently allowed the calculation of fat-free mass [36]. This method, based on Archimedes’ principle, determines body volume by measuring water displacement during submersion and has historically been regarded as a standard for FFM assessment [37]. Although hydrodensitometry offers high accuracy under controlled conditions, it requires substantial subject cooperation, is technically demanding, and may not be suitable for all populations, such as young children or elderly individuals [36,38].

Another method occasionally utilized was whole-body potassium counting, which provides an indirect estimation of body cell mass by measuring the natural radioactive decay of potassium-40 in body tissues [39]. Although this technique allows direct inference of cellular mass, its application is limited due to the need for specialized equipment and the complexity of the measurement procedure [39].

Some studies also used air displacement plethysmography (ADP) as a reference method for body composition assessment. ADP, based on the measurement of body volume through air displacement, enables the estimation of body density, from which fat-free mass can be derived [40]. This method, often performed using devices such as the BOD POD^®^, offers a less invasive and more tolerable alternative to hydrodensitometry, particularly beneficial in paediatric or mobility-impaired populations [40].

The incorporation of these alternative reference methods, although less frequent, contributed to the diversity of prediction equations included in this review and supported the validation of models estimating parameters such as FFM and BCM.

## Description of estimated parameters

The application of bioelectrical impedance analysis for body composition assessment relies on the estimation of specific physiological compartments based on measured impedance values and derived prediction equations. Each estimated parameter reflects distinct biological and clinical characteristics, and the accuracy of their prediction is essential for the validity of BIA-based assessments. In the present review, six primary body composition parameters were analysed: total body water, extracellular water, intracellular water, fat-free mass, body cell mass, and bone mineral content. This section provides a brief description of each estimated parameter, highlighting its physiological relevance, its role in body composition analysis, and its methodological implications as observed in the reviewed studies.

## Total body water (TBW)

Total body water constitutes the largest single component of body composition, accounting for approximately 50–70 % of body weight depending on age, sex, and body composition [41]. TBW is distributed between two major compartments: intracellular water, representing the water contained within cells, and extracellular water, encompassing plasma, interstitial fluid, and transcellular fluids [42].

The estimation of TBW using BIA is based on the principle that water-containing tissues conduct electrical current more readily than tissues with low water content, such as fat mass. Consequently, impedance measurements, particularly resistance, are inversely related to the amount of conductive fluid present in the body [43]. In multi-frequency BIA, lower frequencies (<50 kHz) are primarily sensitive to ECW, while higher frequencies penetrate cell membranes, allowing the estimation of total body water [44].

In the reviewed studies, TBW was one of the most frequently targeted parameters for prediction equation development. Techniques such as deuterium dilution were commonly employed as reference methods to validate BIA-derived TBW estimates, ensuring high accuracy [16,20,28]. Studies consistently highlighted the importance of using standardized measurement protocols, including electrode placement, body position, and fasting status, to minimize variability in TBW estimation [41,45].

Given the critical role of TBW in reflecting hydration status, nutritional state, and overall health, accurate estimation of this compartment is essential for clinical practice and research applications. The development of robust prediction equations for TBW assessment remains a key focus in advancing the utility of BIA technologies [20,28,45].

## Extracellular water (ECW)

Extracellular water refers to the portion of total body water located outside the cells, including plasma, interstitial fluid, and transcellular compartments such as cerebrospinal and synovial fluids [42,46]. ECW consti-tutes a constant proportion of total body water under normal physiological conditions, although this proportion can vary depending on factors such as age, sex, and health status [42,47].

In BIA, the estimation of ECW is facilitated by the use of low-frequency electrical currents, which are unable to penetrate cell membranes and thus travel predominantly through the extracellular space [48]. Multi-frequency BIA devices exploit this frequency-dependent behaviour to separately estimate ECW and ICW by analysing impedance at various frequencies [44].

Among the studies included in this review, only a limited number developed prediction equations specifically targeting ECW. This is partly due to the technical challenges in distinguishing ECW from ICW with high precision and the limited availability of direct reference methods for ECW measurement [48,49]. Deuterium dilution combined with sodium bromide dilution has occasionally been used as a reference for validating ECW estimates, although this approach is complex and not routinely applied [42].

Accurate assessment of ECW is clinically significant, particularly in monitoring conditions associated with fluid imbalance such as oedema, dehydration, and certain chronic diseases. Developing reliable BIA-based models for ECW estimation thus remains a challenging but important area of research [46,49].

## Intracellular water (ICW)

Intracellular water constitutes the portion of total body water located within the cells, playing a critical role in maintaining cellular structure, metabolism, and biochemical reactions [42,50]. In healthy individuals, ICW generally accounts for approximately two-thirds of total body water, although this ratio can be influenced by age, sex, body composition, and health status [42].

The estimation of ICW using BIA relies on the application of multi-frequency electrical currents. At higher frequencies, the electrical current is capable of penetrating cell membranes, allowing the assessment of the conductive properties of intracellular compartments [44,51]. Multi-frequency BIA thus enables the separate estimation of ECW and ICW by analysing impedance responses across a range of frequencies.

In the reviewed studies, direct validation of ICW estimates was less common compared to TBW or FFM. This is partly because there is no single universally accepted direct reference method for measuring ICW. Some studies used mathematical subtraction methods, deriving ICW values by calculating the difference between TBW (measured by deuterium dilution) and ECW (measured by sodium bromide dilution) [42,48]. Despite these methodological challenges, accurate estimation of ICW is important in clinical practice, particularly for evaluating conditions such as cellular dehydration, malnutrition, and certain disease states associated with fluid redistribution [50,51].

## Fat-free mass (FFM)

Fat-free mass includes all body components excluding fat, comprising mainly water, protein, minerals, and glycogen [43,52]. It is a physiologically important compartment, representing the metabolically active tissues of the body and serving as a key determinant of basal metabolic rate, physical strength, and functional capacity [53].

The estimation of FFM using BIA is based on the principle that the electrical conductivity of the body is predominantly determined by the water and electrolyte content of lean tissues. As fat tissue is relatively poor in water and electrolytes, it offers greater resistance to electrical current, whereas lean tissue conducts current more easily [43,54]. Therefore, resistance and reactance values measured by BIA, particularly at a frequency of 50 kHz, have been widely used to predict FFM [44].

Many of the studies included in this review focused on the development of prediction equations for FFM, often using DXA as a reference method for validation [28,21,29]. In some cases, multi-component models incorporating measurements of body density, total body water, and bone mineral content were employed to derive more accurate estimates of FFM [28,34]. These studies emphasized the importance of accounting for population-specific characteristics, such as age, sex, and ethnicity, when developing and applying prediction models, as FFM distribution varies significantly among different demographic groups [53,55].

Given the central role of FFM in health and disease, the development of accurate BIA-based prediction equations for this parameter remains a primary focus in both clinical and research contexts [52,55].

## Body cell mass (BCM)

Body cell mass represents the metabolically active component of fat-free mass, encompassing the mass of all cells capable of performing biological work, including muscle cells, organ tissue, blood cells, and immune cells [50,56]. BCM is crucial for maintaining vital physiological functions such as energy metabolism, immune response, and tissue repair.

Estimation of BCM using BIA is more complex compared to parameters like TBW or FFM. BCM is closely related to intracellular water, as most metabolically active cells are located inside the intracellular compartment, which makes ICW a good indicator of BCM [50]. Some studies derive BCM indi-rectly by applying formulas based on ICW measu-rements, assuming a relatively constant relationship between cellular hydration and cell mass [42,48]. Specific prediction models for BCM have been developed in a limited number of studies, often using reference techniques such as whole-body potassium counting or multi-component models that integrate multiple body composition measurements [34,39].

Accurate assessment of BCM is clinically important, particularly in conditions associated with catabolic states, such as malnutrition, chronic diseases, and cachexia, where a decline in BCM serves as a critical marker of disease severity and prognosis [56,57]. However, due to the challenges in direct measurement and the dependence on indirect estimation methods, the development of reliable BIA-based equations for BCM remains an area requiring further research and refinement.

## Bone mineral content (BMC)

Bone mineral content reflects the total amount of mineral substances contained within the skeletal system, primarily calcium and phosphorus [27,58]. BMC is a critical indicator of bone strength and structural integrity, and it plays a key role in the assessment of growth, development, and bone-related disorders such as osteoporosis [58].

In bioelectrical impedance analysis, direct estimation of BMC is more challenging compared to other compartments because bone tissue has different electrical properties compared to soft tissues [59]. Nevertheless, some multi-frequency BIA models attempt to estimate BMC indirectly by incorporating parameters such as reactance and resistance at specific frequencies, body geometry, and overall body composition data [59]. The reviewed studies developing BMC prediction equations frequently utilized dual-energy X-ray absorptiometry as the reference method, given its ability to provide precise and region-specific measurements of bone mass [28,60].

Although the estimation of BMC by BIA is still less standardized than TBW or FFM prediction, it represents an emerging area of research with the potential to expand the clinical utility of BIA beyond traditional applications [58,59]. Accurate prediction of BMC could be especially valuable for screening and monitoring bone health in populations where access to DXA is limited.

## Results

A total of 43 studies were included in this review, leading to the identification of 98 unique prediction equations for six primary body composition parameters: total body water, extracellular water, intracellular water, fat-free mass, body cell mass, and bone mineral content. A detailed summary of all 98 prediction equations, including estimated parameters, study populations, reference methods, and validation statistics, is presented in Supplementary Table 1.

**Fig. 2:**
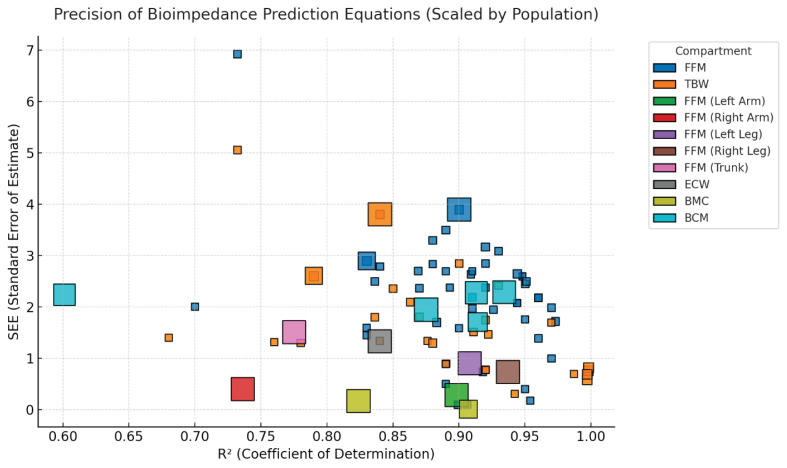
Relationship between the coefficient of determination (R^2^) and the standard error of estimate (SEE) for all identified bioimpedance prediction equations. Square size represents study sample size, and colour distinguishes the estimated body compartment. Equations with high R^2^ and low SEE may be found toward the lower right region of the plot, indicating greater predictive accuracy and reliability.

Overall, the coefficients of determination (R^2^) and standard errors of estimate (SEE) reported across the identified equations demonstrate a wide range of predictive accuracy depending on the estimated parameter and study design. Most equations achieved R^2^ values above 0.80, indicating strong model performance, while SEE values varied substantially between compartments and reference methods. Equations predicting total body water and fat-free mass generally showed the highest consistency and precision, whereas those targeting smaller or more specific compartments, such as body cell mass and bone mineral content, exhibited greater variability. The relationship between R^2^ and SEE, along with the influence of study sample size, is illustrated in Figure 2.

**Fig. 3:**
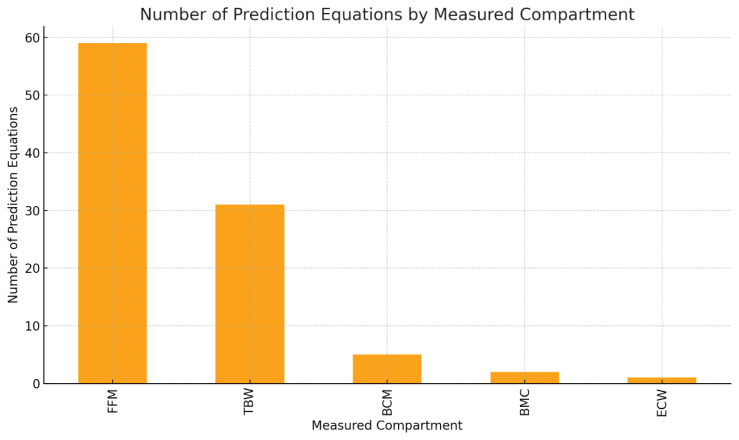
Number of prediction equations developed for each body composition compartment.

Regarding the distribution of prediction equations across parameters, fat-free mass was the most commonly targeted compartment, with a total of 59 developed equations. Total body water was the second most frequently estimated parameter, with 31 equations. In contrast, only two equations were developed for bone mineral content, five equations for body cell mass, and one equation for extracellular water. No original prediction equation for intracellular water was identified in the included studies. The number of prediction equations corresponding to each compartment is summarized in Figure 3.

**Fig. 4:**
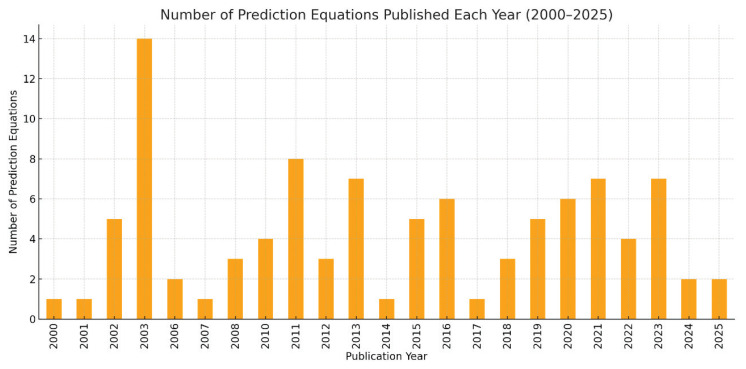
Number of prediction equations published each year between 2000 and 2025.

The temporal distribution of published prediction equations shows notable variability across the examined period. The earliest surge in model development was observed in 2002 and 2003, with 5 and 14 new equations published, respectively. This initial peak was followed by periods of moderate activity, with notable outputs in 2011 (8 equations) and 2013 (7 equations). In contrast, years such as 2007 and 2014 showed only minimal activity, each contributing a single new equation. Between 2016 and 2019, the number of new equations stabilized at a moderate level, ranging from 5 to 6 annually. After 2020, a mild resurgence in publication activity was observed, with 6 to 7 new equations developed per year, potentially reflecting renewed research interest and technological advancements in multi-frequency BIA devices. Overall, the data indicate fluctuating but sustained interest in BIA-based body composition modelling over the past two decades. The distribution of equations across publication years is illustrated in Figure 4.

Most of the included studies employed either deuterium dilution or dual-energy X-ray absorptiometry as reference methods for model validation. A smaller subset of studies used multi-component models, hydrodensitometry, or air displacement plethysmography. This reference methods varied based on the specific parameter being predicted and the available measurement technologies at the time of each study.

## Discussion

This review analysed the development of bioelectrical impedance analysis prediction equations published between 2000 and 2025, focusing on total body water, extracellular water, intracellular water, fat-free mass, body cell mass, and bone mineral content. The findings highlight notable patterns in research activity, methodological choices, and areas requiring further investigation.

The majority of identified prediction equations targeted FFM and TBW, reflecting the longstanding clinical and research interest in these compartments. In contrast, parameters such as ECW, ICW, BCM, and BMC received considerably less attention, as evidenced by the limited number of developed equations. This trend suggests that FFM and TBW are regarded as more feasible or clinically relevant compartments for estimation using BIA, while technical challenges related to measuring ECW, ICW, and BMC may have hindered broader development efforts.

The methodological quality of the included studies was generally acceptable, particularly given the widespread use of deuterium dilution and dual-energy X-ray absorptiometry as reference standards. The reliance on these established methods enhances confidence in the validity of many developed equations. However, it should be noted that multi-component models, although offering higher precision, were underutilized, likely due to their complexity and resource demands. The heterogeneity in reference methods, along with differences in sample populations, further complicates direct comparisons between prediction equations derived from different studies.

Patterns in publication activity revealed an early surge in model development during 2002 and 2003, coinciding with an expanding application of BIA technologies in clinical and research settings. Subsequent fluctuations in publication rates, with notable outputs in 2011 and 2013 and a modest resurgence after 2020, likely reflect shifts in research funding priorities, technological advancements, and growing emphasis on personalized healthcare. However, despite sustained interest, the overall number of new prediction equations remains modest compared to the expanding global application of BIA, indicating ongoing opportunities for methodological innovation and model refinement.

Analysis of the compiled prediction equations further highlights specific trends. Among the reviewed models, Sun *et al*. [5] contributed substantially to FFM estimation based on multicomponent models used in large epidemiological cohorts. Bedogni *et al*. [80] provided important validation work for TBW estimation using an eight-point electrode approach. On the other hand, compartments such as BCM and BMC were addressed less frequently, with Pietrobelli *et al*. [58] proposing an advanced impedance approach for BMC estimation. Despite technical advances, no new validated ICW-specific prediction equations were identified, underscoring the persistent challenges in intracellular fluid assessment [5,42].

Moreover, while traditional 50 kHz single-frequency BIA remains prevalent, a shift toward multi-frequency BIA technologies is evident in newer models aiming to improve differentiation between ECW and ICW [6,17]. This methodological shift reflects the advantage of multi-frequency measurements in resolving body water compartments, particularly relevant for clinical monitoring of hydration and disease-related fluid shifts. Nevertheless, the majority of these equations were developed and validated in healthy adult populations, with only limited application in paediatric, elderly, or clinical groups, thereby restricting their generalizability across broader demographics [14,39].

Several limitations were evident across the reviewed studies. Geographical concentration of the research, primarily within North America, Europe, and parts of Asia, limits the generalizability of many prediction equations to broader global populations. Furthermore, many studies focused on healthy adults, with relatively fewer models developed specifically for paediatric, elderly, or clinical populations characterized by altered fluid distribution or disease-related body composition changes. The limited availability of validated equations for ECW, ICW, BCM, and BMC further highlights gaps in the current literature, underscoring the need for future research to expand beyond traditional compartments.

Moreover, differences in measurement protocols have been shown to significantly influence impedance values and thereby affect the accuracy of prediction equations. Previous studies have systematically analysed the impact of various procedural factors, including fasting status, hydration, voiding, recent physical activity, body position, ambient temperature, electrode placement, and device calibration. Food and beverage consumption may decrease impedance by 4–15 Ω within a few hours after meals, while dehydration can increase resistance by approximately 40 Ω, resulting in an underestimation of fat-free mass by about 5 kg. Exercise has been reported to reduce resistance by around 3 % and reactance by 8 %, with values normalizing after one hour of rest. Body position is also critical, as subjects should remain supine for 4–10 min before measurement to allow fluid stabilization; prolonged recumbency may further lower resistance due to extracellular fluid redistribution. Correct electrode placement with a minimum inter-electrode distance of 5 cm, consistent measurement on the same body side, and avoidance of contact with conductive or metallic surfaces are essential to ensure accurate current flow. Ambient temperature and skin surface temperature additionally influence impedance, while bladder voiding before measurement can minimize error to approximately 1 %. These findings consistently emphasize the importance of standardized measurement conditions and device calibration to improve the reproducibility and comparability of BIA-derived prediction equations [10,94].

Overall, this review brings together more than two decades of research on multi-frequency BIA prediction equations, creating an updated and comprehensive evidence base. It identifies validated models, methodological patterns, and gaps in current knowledge, thereby supporting both scientific progress and clinical application. By outlining underrepresented populations and parameters, the review assists researchers and practitioners in selecting appropriate equations, designing robust validation protocols, and developing new models suited to modern devices and diverse cohorts. In this way, the review contributes to improving the reliability and interpretability of bioimpedance-based measurements and supports their more effective use in clinical evaluation, nutritional monitoring, and public health research.

Finally, technological advances in multi-frequency BIA devices and modelling techniques offer promising avenues for improving prediction accuracy. The development of population-specific equations, incorporation of broader demographic characteristics, and external validation in diverse clinical settings are essential steps to ensure the applicability and robustness of future BIA models.

## Supplementary Information
