# Supplementary material for: Multi-Frequency Bioimpedance Analysis in Practice: A Review of Validated Prediction Equations for Key Body Composition Parameters
Source: Physiol Res. 2025 Dec 1;74(Suppl 1):S77–92. doi: 10.33549/physiolres.935758 (PMC12849772; doi:10.33549/physiolres.935758)
Supplement: Supplementary file 1 [file PR74_S77_Suppl_Table_1.pdf]

Supplementary Table 1. BIA Prediction Equations by Measured Compartment.

Estimated Compartment	Author [Ref]	N (M/F)	Age Range	Population	BIA Device	Reference Method	Equation	Parameters	R² / SEE	Electrode Placement
BCM	Pirlich et al. [23]	19 (14/5)	Adult (mean ~47)	German adults (healthy controls); Healthy controls	BIA 2000-M, Data Input GmbH (50 kHz)	Total-body potassium (TBK)	BCM = 0.534×(H/Rwhole-body) + 0.233×Xcwhole-body - 19.25	Height (m), Resistance (Ohm), Reactance (Ohm) at 50 kHz	0.875 / 1.95	Hand-to-foot (tetrapolar)
BCM	Pirlich et al. [23]	17 (10/7)	Adult (mean ~54)	German adults (liver cirrhosis without ascites); Liver cirrhosis without ascites	BIA 2000-M, Data Input GmbH (50 kHz)	Total-body potassium (TBK)	BCM = 0.174×(H/Rwhole-body) + 11.44×ln(jwhole-body) + 0.129×W - 17.11	Height (m), Resistance (Ohm), Phase angle (degrees), Weight (kg) at 50 kHz	0.913 / 2.28	Hand-to-foot (tetrapolar)
BCM	Pirlich et al. [23]	16 (12/4)	Adult (mean ~53)	German adults (liver cirrhosis with ascites); Liver cirrhosis with ascites	BIA 2000-M, Data Input GmbH (50 kHz)	Total-body potassium (TBK)	BCM = 0.148×(H/Rwhole-body) + 9.98×ln(jwhole-body) - 5.163	Height (m), Resistance (Ohm), Phase angle (degrees) at 50 kHz	0.601 / 2.24	Hand-to-foot (tetrapolar)
BCM	Pirlich et al. [23]	12 (5/7)	Adult (mean ~42)	German adults (Cushing's disease); Cushing's disease	BIA 2000-M, Data Input GmbH (50 kHz)	Total-body potassium (TBK)	BCM = 0.172×Xcwhole-body + 42.353×H - 61.287	Height (m), Reactance (Ohm) at 50 kHz	0.914 / 1.71	Hand-to-foot (tetrapolar)
BCM	Pirlich et al. [23]	18 (12/6)	Adult (mean ~50)	German adults (acromegaly); Acromegaly	BIA 2000-M, Data Input GmbH (50 kHz)	Total-body potassium (TBK)	BCM = 0.373×(H/Rwhole-body) + 0.447×Xcwhole-body + 0.156×W - 30.499	Height (m), Resistance (Ohm), Reactance (Ohm), Weight (kg) at 50 kHz	0.934 / 2.29	Hand-to-foot (tetrapolar)
BMC	Patil et al. [6]	113 (28/85)	23–81	Indian adults; Healthy adults	InBody 720 (Biospace), segmental MF-BIA	DXA	BMC = -0.243 - 634×(h²/Zbody250) - 0.0159×Age + 5.79×h - 0.0041×Rbody250 + 0.048×(Xbody250/h) - 0.05×((Zbody5 - Zbody250)/Age) - 0.48×Φbody250	Height (m), Zbody250 (Ohm), Age (years), Rbody250 (Ohm), Xbody250 (Ohm), Zbody5 (Ohm), Φbody250 (degrees); 250 kHz and 5 kHz.	0.8237 / 0.168	Foot-to-hand (standing, segmental)
BMC	de Castro et al. [91]	64 (29/35)	8–15	Brazilian children and adolescents with HIV; HIV-infected children and adolescents	InBody 720 (Biospace), octapolar, 50 kHz	DXA	BMC = -0.8134 + 0.0452×Body Mass - 0.0149×Fat Mass BIA + 0.0044×Total Reactance - 0.0002×TCD4 - 0.0615×ART type + 0.0123×%TCD4	Body Mass (kg), Fat Mass measured by BIA (kg), Total Reactance (Ohm at 50 kHz), Total CD4+ lymphocyte count (cells/μL), ART type (0 = without protease inhibitor, 1 = with protease inhibitor), Percentage of CD4+	0.907 / 0.011	Foot-to-hand (octapolar)

								lymphocytes (%); 50 kHz.		
ECW	Matias et al. [2]	208 (138/70)	18–30 (mean ~21)	National-level Portuguese athletes; Healthy, national-level athletes	BIA-101 RJL/Akern Systems (50 kHz)	Bromide dilution	ECW = 1.579 + 0.055×Stature²/Resistance + 0.127×Weight + 0.006×Stature²/Reactance + 0.932×Sex (Sex: Male=1, Female=0)	Weight (kg), Stature (cm), Resistance (Ohm), Reactance (Ohm); 50 kHz	0.84 / 1.33	Hand-to-foot
FFM	Aglago et al. [69]	250 (56/194)	18–64 years	Moroccan, North-African adults; Healthy adults	Quadscan 4000 (Bodystat, Isle of Man, UK)	Deuterium dilution (²H□O)	FFM (kg) = 7.47 + 0.366 × (Height²/Resistance) + 6.04 × Sex + 0.306 × Weight - 0.063 × Age	Height in cm, Resistance in ohms, Weight in kg, Age in years, Sex (male=1, female=0), measured at 50 kHz	0.92 / 2.38 kg	Tetrapolar (wrist-to-ankle, supine position)
FFM	Aguilar-Troncoso et al. [16]	328 (171/ 157)	10–17 years	Mexico; Healthy children and adolescents	SECA mBCA 515/514 (50 kHz)	4C model (ADP + D□O + DXA)	-15.343 + 0.2081 × Weight + 0.8814 × (Height²/Resistance) + 0.2055 × Reactance	Height in cm, Resistance and Reactance in ohms at 50 kHz, Weight in kg	0.96 / 2.18 kg	hand-to-foot
FFM	Aguilar-Troncoso et al. [61]	60 (29/31)	10–17 years	Mexican (youths); Apparently healthy adolescents	seca mBCA 514	4C model (DXA, BodPod, TBW by D□O, BMC)	FFM (kg) = 0.2081·W + 0.8814·(H²/R) + 0.2055·Xc – 15.343	Weight (kg), Height² (cm²)/Resistance (Ω), Xc in Ohms, 50 kHz	0.96 / 2.18 kg	Hand-to-foot (seca mBCA standard)
FFM	Alemán-Mateo et al. [77]	383 (214/ 169)	60–89 years	Mexico, Guatemala, Brazil, Morocco, Senegal; Older adults from developing countries	RJL Systems BIA-101, Xitron 4000B, Bodystat Quadscan 4000	Deuterium dilution	2.95 - 3.89 × Gender (male=0, female=1) + 0.514 × (Height²/Impedance) + 0.090 × Waist + 0.156 × Weight	Height in cm, Impedance in ohms, Waist in cm, Weight in kg, Gender binary coded (male=0, female=1)	0.88 / 3.3 kg	hand-to-foot
FFM	Caicedo-Eraso et al. [49]	60 (0/60)	18–24 years	Colombia (adult women); Healthy young adult women	Hydra 4200 (Xitron Technologies)	Deuterium dilution (D□O) + Hydrodensitometry	FFM (kg) = 0.774 × (Height²/Resistance50) + 0.204 × Weight + 0.025 × Resistance50 - 18.081	Height in cm, Resistance at 50 kHz in ohms, Weight in kg, measured at 50 kHz	0.9 / 1.59 kg	Tetrapolar (wrist-to-ankle)
FFM	Campa et al. [25]	102 (102/0)	18–35 (mean 24.7 ± 5.7)	Italian Serie A soccer players; Healthy elite athletes	BIA 101 BIVA®PRO (Akern Systems)	DXA	FFM = -7.729 + (0.686×Body Mass) + (0.227×Stature²/Resistance) + (0.086×Reactance) + (0.058×Age)	Body Mass (kg), Stature (cm), Resistance (Ohm), Reactance (Ohm), Age (years); 50 kHz	0.97 / 1	Foot-to-hand
FFM	Cattem et al. [48]	149 (149/0)	13–19 years	Brazil (multiracial, 'pardos'); Healthy adolescent soccer athletes	SECA mBCA-515 (50 kHz)	DXA (Lunar iDXA, GE Healthcare)	-7.064 + 0.592 × Age + 0.554 × Weight + 0.365 × (Height²/Resistance)	Age in years, Height in cm, Resistance in ohms at 50 kHz, Weight in kg	0.95 / 1.76 kg	hand-to-foot

FFM	Chumlea et al. [3]	15903 (7716/ 8187)	12–80 years	USA (NH White, NH Black, Mexican-American); General US population sample	Valhalla 1990B (converted to RJL equivalent)	Isotope dilution (^{2}H)	FFM (kg) = -10.678 + 0.262·weight + 0.652·(height^2 / resistance) + 0.015·resistance	Height^2 in cm^2, weight in kg, resistance at 50 kHz	0.9 / 3.90 kg	Hand-to-foot (tetrapolar)
FFM	Chumlea et al. [3]	8187 (0/8187)	12–80 years	USA (NH White, NH Black, Mexican-American); General US population sample	Valhalla 1990B (converted to RJL equivalent)	Isotope dilution (^{2}H)	FFM (kg) = -9.529 + 0.168·weight + 0.696·(height^2 / resistance) + 0.016·resistance	Height^2 in cm^2, weight in kg, resistance at 50 kHz	0.83 / 2.90 kg	Hand-to-foot (tetrapolar)
FFM	Costa et al. [83]	257 (129/ 128)	10–19 years	Brazil; Healthy adolescents	Biodynamics model 450 (50 kHz)	DXA (Lunar Prodigy Advance GE Healthcare)	-17.189 + 0.498 × (Height^2/Resistance) + 0.226 × Weight + 0.071 × Reactance - 2.378 × Sex (male=0, female=1) + 0.097 × Height + 0.222 × Age	Height in cm, Weight in kg, Resistance and Reactance in ohms at 50 kHz, Age in years, Sex binary coded (male=0, female=1)	0.951 / 2.5 kg	hand-to-foot
FFM	Dey et al. [21]	106 (51/55)	75 years	Sweden; Healthy elderly	RJL Systems BIA-101 (50 kHz)	4C model (TBK + TBW)	11.78 + 0.499 × (Height^2/Resistance) + 0.134 × Weight + 3.449 × Sex (male=1, female=0)	Height in cm, Resistance in ohms at 50 kHz, Weight in kg, Sex binary coded (male=1, female=0)	0.909 / 2.64 kg	hand-to-foot
FFM	Divala et al. [13]	186 (79/107)	10–18 years	Malawi; Healthy adolescents	Bodystat 1500 MDD (Bodystat Ltd)	Deuterium dilution (DDM)	FFM (kg) = -4.316 + 0.425 × (Height^2/Resistance) + 1.287 × Sex + 0.307 × Age + 0.344 × Weight + 0.019 × Reactance	Height in cm, Resistance in ohms, Weight in kg, Age in years, Sex (male=1, female=0), Reactance in ohms, measured at 50 kHz	0.926 / 1.95 kg	Tetrapolar (wrist-to-ankle)
FFM	El Harchaoui et al. [19]	247 (122/ 125)	8–11 years	Morocco; Healthy school-aged children	Bodystat QuadScan 4000 (50 kHz)	Deuterium dilution	-0.450 + 0.380 × (Height^2/Resistance) + 0.291 × Weight + 0.446 × Age + 1.294 × Sex (male=1, female=0)	Height in cm, Resistance in ohms at 50 kHz, Weight in kg, Age in years, Sex coded (male=1, female=0)	0.91 / 1.971 kg	hand-to-foot
FFM	Haapala et al. [79]	93 (0/93)	62–72 years	Finland; Healthy elderly women	RJL Systems BIA-101 (50 kHz)	DXA	-128.06 + 1.85 × BMI - 0.63 × Weight + 1.07 × Height - 0.03 × Resistance + 10.0 × Waist-to-Hip Ratio	BMI in kg/m^2, Height in cm, Weight in kg, Resistance in ohms at 50 kHz, Waist-to-hip ratio as dimensionless value	0.83 / 1.6 kg	hand-to-foot
FFM	Hastuti et al. [70]	292 (292/0)	18–65 years	Indonesian (Javanese ethnicity); Healthy adult men	Imp DF50 (ImpediMed Ltd., Australia)	Deuterium dilution (^{2}H□O)	FFM (kg) = 0.467 + 0.291 × Body Weight + 0.448 × Resistance Index + 0.072 × Reactance	Body Weight in kg, Resistance Index (Height^2/Resistance) with Height in cm and Resistance in ohms, Reactance in ohms, measured at 50 kHz	0.836 / 2.498 kg	Tetrapolar (wrist-to-ankle, supine position)
FFM	Hofsteenge et al. [88]	310 (124/ 186)	11–18 years	Dutch (Netherlands); Obese adolescents	Xitron Hydra 4200 (50 kHz)	DXA (Hologic Delphi A)	0.527 × (Height^2/Impedance) + 0.306 × Weight - 1.862	Height in cm, Weight in kg, Impedance in ohms at 50 kHz	0.92 / 2.85 kg	hand-to-foot

FFM	Jayawardena et al. [44]	170 (56/114)	19–69 years	Sri Lanka; Healthy adults	InBody 230 (multi-frequency analyzer)	Deuterium dilution	-30.188 + 0.593 × (Height^2/Resistance) + 3.206 × Gender (male=1, female=0) + 0.130 × WC + 0.151 × HC + 0.178 × Height	Height, WC, HC in cm; Resistance in ohms; Gender binary coded (male=1, female=0)	0.732 / 6.93 kg	hand-to-foot
FFM	Kanellakis et al. [86]	694 (265/ 429)	18–80 years	Greek (Caucasian); Healthy adults	Akern BIA101 (50 kHz)	DXA	12.299 + 0.164 × Weight + 7.287 × Gender (male=1, female=0) - 0.116×(Resistance/Height^2) + 0.365 × (Reactance/Height^2) + 21.570 × Height	Height in meters, Weight in kg, Resistance and Reactance in ohms at 50 kHz, Gender coded (male=1, female=0)	0.944 / 2.65 kg	hand-to-foot
FFM	Khan AI et al. [72]	200 (102/98)	4–10 years	Bangladeshi children; Healthy rural children	Tanita TBF-300A (Tanita Corporation, Tokyo, Japan)	Deuterium dilution (^{2}H□O)	FFM (kg) = 2.34 + 0.50 × Weight - 0.52 × Sex + 0.18 × Age + 0.21 × (Height^2/Impedance)	Weight in kg, Height in cm, Impedance in ohms, Age in years, Sex (male=1, female=2), measured at 50 kHz	0.89 / 0.90 kg	Leg-to-leg (standing)
FFM	Kourkoumelis et al. [18]	266 (143/ 123)	7.3–14.5 years	Europe (Bosnia and Herzegovina, Latvia, Montenegro, North Macedonia, Portugal); Healthy children and adolescents	Impedimed DF50 and BodyStat 1500MDD (50 kHz)	Deuterium dilution	FFM = 0.59 × (Height^2/Resistance) + 0.16 × Weight + 0.71 × Sex (male=1, female=0) + 1.4	Height in cm, Resistance in ohms at 50 kHz, Weight in kg, Sex binary coded	0.87 / 2.37 kg	hand-to-foot
FFM	Kyle et al. [22]	343 (202/ 141)	22–94 years	Switzerland (white Europeans); Healthy ambulatory adults	Xitron 4000B (50 kHz)	DXA (Hologic QDR-4500)	-4.104 + 0.518 × (Height^2/Resistance) + 0.231 × Weight + 0.130 × Reactance + 4.229 × Sex (male=1, female=0)	Height in cm, Resistance and Reactance in ohms at 50 kHz, Weight in kg, Sex binary coded (male=1, female=0)	0.973 / 1.72 kg	hand-to-foot
FFM	Lazzer et al. [80]	58 (27/31)	10–17 years	Italy (Caucasian children); Severely obese children and adolescents	Human IM Plus II (DS Medica, multifrequency 5–250 kHz)	DXA	0.87 × (Height^2/Impedance) + 3.1	Height in cm, Impedance in ohms, measured with multifrequency BIA	0.91 / 2.7 kg	hand-to-foot
FFM	Lewis et al. [17]	50 (27/23)	12–59 months	Ugandan; Stunted children (HAZ < -2), non-SAM, no medical complications	Bodystat 500	Deuterium dilution (^{2}H)	FFM (kg) = 3.796 + 0.488·sex + 0.068·age + 0.400·(H^2/Z_{50}) + 0.214·HAZ	Height^2 in cm^2, impedance at 50 kHz, age in months, sex (male=1, female=0), HAZ (Z-score)	0.95 / 0.402 kg	Hand-to-foot (tetrapolar, right side)
FFM	Lingwood et al. [87]	77 (41/36)	Birth (0–3 days)	Australia (Caucasian neonates); Healthy neonates	ImpediMed SFB7 (multi-frequency BIA)	PEA POD (air displacement plethysmography)	0.779 + 0.655 × Weight - 0.072 × Sex (male=1, female=2) + 0.037 × (Length^2/$Resistance_0$)	Length in cm, Weight in kg, Resistance in ohms ($Resistance_0$), Sex coded (male=1, female=2)	0.94 / nan	Supine (hand-to-foot)
FFM	Lingwood et al. [87]	54 (nan/ nan)	6 weeks	Australia (Caucasian neonates); Healthy neonates	ImpediMed SFB7 (multi-frequency BIA)	PEA POD (air displacement plethysmography)	1.169 + 0.568 × Weight - 0.128 × Sex (male=1, female=2) + 0.032 × (Length^2/$Resistance_0$)	Length in cm, Weight in kg, Resistance in ohms ($Resistance_0$), Sex coded (male=1, female=2)	0.89 / nan	Supine (hand-to-foot)

FFM	Lingwood et al. [87]	55 (nan/ nan)	3 months	Australia (Caucasian neonates); Healthy neonates	ImpediMed SFB7 (multi-frequency BIA)	PEA POD (air displacement plethysmography)	1.315 + 0.449 × Weight - 0.169 × Sex (male=1, female=2) + 0.153 × (Length^2/Resistance$_0$)	Length in cm, Weight in kg, Resistance in ohms (Resistance$_0$), Sex coded (male=1, female=2)	0.92 / nan	Supine (hand-to-foot)
FFM	Lingwood et al. [87]	53 (nan/ nan)	4.5 months	Australia (Caucasian neonates); Healthy neonates	ImpediMed SFB7 (multi-frequency BIA)	PEA POD (air displacement plethysmography)	1.909 + 0.280 × Weight - 0.279 × Sex (male=1, female=2) + 0.305 × (Length^2/Resistance$_0$)	Length in cm, Weight in kg, Resistance in ohms (Resistance$_0$), Sex coded (male=1, female=2)	0.89 / nan	Supine (hand-to-foot)
FFM	Liu et al. [68]	630 (328/ 302)	8–10 years	Chinese, Lebanese, Malaysian, Filipino, Thai; Healthy, prepubertal (Tanner stage 1) children	Imp DF50 (ImpediMed Limited, Australia)	Deuterium dilution (^{2}H□O)	FFM (kg) = 0.299 × (Height^2/Resistance) + 0.086 × Height + 0.245 × Weight + 0.260 × Age + 0.901 × Sex - 0.415 × Ethnicity (Thai) - 6.952	Height in cm, Resistance in ohms, Weight in kg, Age in years, Sex (male=1, female=0), Ethnicity dummy (Thai=1, others=0), measured at 50 kHz	0.883 / 1.7 kg	Tetrapolar (wrist-to-ankle, right side)
FFM	Luke et al. [14]	362 (176/ 186)	25–45 years	African origin (Ghana, South Africa, Jamaica, Seychelles, USA); Healthy adults	RJL Systems BIA 101Q (50 kHz)	Doubly labeled water (isotope dilution)	12.6 + 0.22 × Weight + 0.46 × (Height^2/Resistance) - 5.7 × Sex (male=0, female=1)	Weight in kg, Height in cm, Resistance in ohms at 50 kHz, Sex binary coded (male=0, female=1)	0.89 / 3.5 kg	hand-to-foot (supine position)
FFM	Macias et al. [78]	155 (73/82)	20–50 years	Mexico; Healthy adults	RJL Systems BIA-103 (50 kHz)	Air Displacement Plethysmography (ADP, Bod Pod)	0.7374 × (Height^2/Resistance) + 0.1763 × Weight - 0.1773 × Age + 0.1198 × Reactance - 2.4658	Height in cm, Resistance and Reactance in ohms at 50 kHz, Weight in kg, Age in years	0.97 / 1.99 kg	hand-to-foot
FFM	Masset et al. [31]	178 (přibližně 58/přibližně 120)	18–65 years	Brazilian ; Overweight or obese adults	Bodystat 1500 (50 kHz)	DXA (GE Lunar Prodigy Advance)	-8.395 + 0.340 × (Height^2/Resistance) - 5.760 × Sex + 0.222 × Weight - 0.041 × Age + 0.138 × Height + 0.700 × PhA	Height in cm, Weight in kg, Resistance and Phase Angle at 50 kHz, Age in years, Sex coded (male=0, female=1)	0.89 / 2.70 kg	hand-to-foot
FFM	Masset et al. [31]	91 (přibližně 30/přibližně 61)	18–65 years	Brazilian; Obese adults	Bodystat 1500 (50 kHz)	DXA (GE Lunar Prodigy Advance)	-0.058 + 0.463 × (Height^2/Resistance) - 5.730 × Sex + 0.278 × Weight + 0.115 × Xc - 0.049 × Age	Height in cm, Weight in kg, Resistance and Reactance at 50 kHz, Age in years, Sex coded (male=0, female=1)	0.88 / 2.84 kg	hand-to-foot
FFM	Matias et al. [50]	66 (66/0)	18+ (mean 23.4 ± 5.4 years)	Portugal; Male elite futsal players (professional and semi-professional)	BIA 101 BIVA PRO (Akern, 50 kHz)	DXA (Hologic Horizon WI)	-8.865 + 0.437 × Body Mass + 0.186 × Xc + 0.415 × (Stature^2/Resistance)	Stature in cm, Resistance and Reactance (Xc) in ohms at 50 kHz, Body Mass in kg	0.893 / 2.38 kg	hand-to-foot
FFM	Nguyen et al. [46]	119 (nan/ nan)	4–7 years	Vietnam; Healthy Vietnamese children	Seca mBCA 525 (50 kHz)	DXA (Hologic Discovery)	0.008 × Age (months) - 0.638 × Sex (male=1, female=0) + 0.189 × Height + 0.167 × Weight - 0.007 × Resistance - 5.539	Age in months, Height in cm, Weight in kg, Resistance in ohms at 50 kHz, Sex binary coded (male=1, female=0)	0.918 / 0.74 kg	hand-to-foot

FFM	Ramel et al. [9]	41 (41/0)	65+ years	Iceland; Healthy elderly	Bodystat 1500 (50 kHz)	DXA (Hologic QDR-2000 Plus)	7.610 - 0.0855 × Age + 0.273 × Weight + 0.148 × Height - 0.00746 × Resistance + 7.998	Age in years, Height in cm, Weight in kg, Resistance in ohms at 50 kHz	0.948 / 2.6 kg	hand-to-foot
FFM	Ramel et al. [9]	57 (0/57)	65+ years	Iceland; Healthy elderly	Bodystat 1500 (50 kHz)	DXA (Hologic QDR-2000 Plus)	7.610 - 0.0855 × Age + 0.273 × Weight + 0.148 × Height - 0.00746 × Resistance	Age in years, Height in cm, Weight in kg, Resistance in ohms at 50 kHz	0.948 / 2.6 kg	hand-to-foot
FFM	Ramírez et al. [67]	167 (87/80)	6–14 years	Mexican (indigenous and non-indigenous children); Healthy children	Impedimed DF50 (Impedimed Pty. Ltd, San Diego, USA)	Deuterium dilution ($^{2}H_{2}O$)	FFM (kg) = 0.661 × (Height^2/Resistance) + 0.200 × Weight - 0.320	Height in cm, Resistance in ohms, Weight in kg, measured at 50 kHz	0.96 / 1.39 kg	Tetrapolar (wrist-to-ankle, supine position)
FFM	Rush et al. [34]	110 (110/0)	19–74 years	Asian Indians (New Zealand); Healthy adults, non-diabetic, physically independent	Impedimed BIM4 (50 kHz)	DXA	0.382 × (Height^2/Resistance) + 0.167 × Weight + 0.320 × Height - 36.382	Height in cm, Resistance in ohms at 50 kHz, Weight in kg	0.84 / 2.79 kg	hand-to-foot
FFM	Rush et al. [34]	101 (0/101)	19–74 years	Asian Indians (New Zealand); Healthy adults, non-diabetic, physically independent	Impedimed BIM4 (50 kHz)	DXA	0.456 × (Height^2/Resistance) + 0.127 × Weight + 0.0746 × Reactance + 5.959	Height in cm, Resistance and Reactance in ohms at 50 kHz, Weight in kg	0.7 / 2.01 kg	hand-to-foot
FFM	Rush et al. [34]	77 (35/42)	22–38 months	Multi-ethnic (New Zealand); Healthy preschool children	BIM4 (Impedimed, 50 kHz)	DXA (GE-Lunar Prodigy)	0.367 × (Height^2/Resistance) + 0.188 × Weight + 0.077 × Height + 0.273 × Sex (male=1, female=0) - 2.490	Height in cm, Resistance in ohms at 50 kHz, Weight in kg, Sex binary coded (male=1, female=0)	0.89 / 0.50 kg	hand-to-foot
FFM	Sluyter et al. [85]	215 (215/0)	12–19 years	New Zealand (multiethnic: European, Maori, Pacific Island, Asian); Healthy adolescents	Tanita BC-418 (50 kHz)	DXA (GE-Lunar Prodigy)	0.607 × (Height^2/Impedance) + 1.542 × Age + 0.220 × Height + 0.096 × Weight + 1.836 × Ethnicity - 47.547	Height in cm, Impedance in ohms at 50 kHz, Weight in kg, Age in years, Ethnicity coded (0 = European or Asian, 1 = Maori or Pacific)	0.93 / 3.09 kg	standing (8-electrode foot-to-hand)
FFM	Sluyter et al. [85]	216 (0/216)	12–19 years	New Zealand (multiethnic: European, Maori, Pacific Island, Asian); Healthy adolescents	Tanita BC-418 (50 kHz)	DXA (GE-Lunar Prodigy)	0.531 × (Height^2/Impedance) + 0.182 × Height + 0.096 × Weight + 1.562 × Ethnicity - 15.782	Height in cm, Impedance in ohms at 50 kHz, Weight in kg, Ethnicity coded (0 = non-Pacific, 1 = Pacific)	0.91 / 2.19 kg	standing (8-electrode foot-to-hand)
FFM	Sun et al. [5]	712 (712/0)	12–94 years	USA (white and black); Healthy adults, broad population sample	Valhalla BIA device, RJL equivalent	Multicomponent model	FFM (kg) = -10.68 + 0.65·(stature^2 / resistance) + 0.26·weight + 0.02·resistance	Height^2 in cm^2, resistance in ohms, weight in kg, single frequency 50 kHz	0.9 / 3.9 kg	Hand-to-foot (tetrapolar)
FFM	Sun et al. [5]	1089 (0/1089)	12–94 years	USA (white and black); Healthy adults, broad population sample	Valhalla BIA device, RJL equivalent	Multicomponent model	FFM (kg) = -9.53 + 0.69·(stature^2 / resistance) + 0.17·weight + 0.02·resistance	Height^2 in cm^2, resistance in ohms, weight in kg, single frequency 50 kHz	0.83 / 2.9 kg	Hand-to-foot (tetrapolar)

FFM	Tint et al. [82]	173 (nan/ nan)	At birth (0 days)	Asian neonates (Singapore); Healthy neonates	ImpediMed SFB7 (multi-frequency including 50 kHz)	Air Displacement Plethysmography (PEA POD)	0.762 × Weight - 0.045 × Sex (male=1, female=0) + 0.010 × (Length²/Resistance$_{50}$) + 0.459	Weight in kg, Length in cm, Resistance at 50 kHz in ohms, Sex binary coded (male=1, female=0)	0.906 / 0.10 kg	hand-to-foot
FFM	Tint et al. [82]	140 (nan/ nan)	2 weeks (±3 days)	Asian neonates (Singapore); Healthy neonates	ImpediMed SFB7 (multi-frequency including 50 kHz)	Air Displacement Plethysmography (PEA POD)	0.621 × Weight - 0.016 × Sex (male=1, female=0) + 0.112 × (Length²/Resistance$_{50}$) + 0.375	Weight in kg, Length in cm, Resistance at 50 kHz in ohms, Sex binary coded (male=1, female=0)	0.899 / 0.096 kg	hand-to-foot
FFM	Valencia et al. [62]	133 (73/60)	>60 years (60–93 years)	Cuba, Chile, Mexico; Healthy elderly, physically independent	RJL Systems BIA-101 (50 kHz)	Deuterium dilution (TBW hydrometry)	-7.71 + 0.49 × (Height²/Resistance) + 1.12 × Country (Chile=1, Mexico=2, Cuba=3) + 0.27 × Weight + 3.49 × Sex (male=1, female=0) + 0.13 × Reactance	Height in cm, Resistance and Reactance in ohms at 50 kHz, Weight in kg, Country coded (Chile=1, Mexico=2, Cuba=3), Sex coded (male=1, female=0)	0.944 / 2.08 kg	hand-to-foot
FFM	Valencia et al. [62]	133 (73/60)	>60 years	Cuba, Chile, Mexico (rural); Healthy older adults	BIA-101 (RJL Systems)	Deuterium dilution (hydrometry)	FFM (kg) = -7.71 + 0.49 × (Height²/Resistance) + 1.12 × Country + 0.27 × Weight + 3.49 × Sex + 0.13 × Reactance	Height in cm, Resistance in ohms, Weight in kg, Reactance in ohms, Country (Chile=1, Mexico=2, Cuba=3), Sex (female=0, male=1), measured at 50 kHz	0.944 / 2.08 kg	Tetrapolar (hand-to-foot)
FFM	Van Zyl et al. [84]	84 (40/44)	5–10 years	South Africa (black children); Healthy school-aged children	Seca mBCA 514 (50 kHz)	DXA (Hologic Discovery W)	105.20 + 0.807 × Sex (male=1, female=0) + 0.174 × Weight + 0.01 × Reactance + 15.71 × log(Height²/Resistance)	Height in cm, Weight in kg, Resistance and Reactance in ohms at 50 kHz, Sex binary coded (male=1, female=0)	0.9544 / nan	Standing (foot-to-hand)
FFM	Wang et al. [32]	255 (127/ 128)	9–19 years	China; Healthy children and adolescents	RJL Systems BIA-101 (50 kHz)	DXA	1.613 + 0.742 × (Height²/Impedance) + 0.151 × Weight	Height in cm, Impedance in ohms at 50 kHz, Weight in kg	0.95 / 2.45 kg	hand-to-foot
FFM	Wibæk et al. [81]	101 (89/97)	0–6 months	Ethiopia; Healthy term infants	Bodystat QuadScan 4000 (50 kHz)	Air Displacement Plethysmography (PEA POD)	1.015 + 0.358 × Weight - 0.132 × Sex (male=0, female=1) + 0.003 × Age (days) + 0.233 × (Length²/Z_{50})	Weight in kg, Sex coded (male=0, female=1), Age in days, Length in cm, Impedance (Z_{50}) in ohms at 50 kHz	0.954 / 0.181 kg	hand-to-foot
FFM	Wickramasinghe et al. [63]	282 (158/ 124)	5–15 years	Sri Lanka; Healthy children	Bodystat (Bodystat Ltd, Isle of Man)	Deuterium dilution (D_2O)	FFM (kg) = 0.56 × (Height²/Impedance) + 0.22 × Weight + 1.6 × Sex - 0.22	Height in cm, Impedance in ohms, Weight in kg, Sex (male=1, female=0), measured at 50 kHz	0.869 / 2.7 kg	Tetrapolar (hand-to-foot)
FFM	Wu et al. [89]	554 (297/ 257)	16–75	Taiwanese (Asian); Healthy individuals	QuadScan 4000 + Omron HBF-361	DXA	FFM = 13.055 + (0.204×Weight) + (0.394×Height²/Impedance) - (0.136×Age) + (8.125×Sex)	Weight (kg), Height (cm), Impedance (Ohm), Age (years), Sex (Male=1, Female=0); 50 kHz	0.92 / 3.17	Foot-to-foot

FFM	Yannakoulia et al. [90]	42 (0/All females)	18–30	Greek professional dancers; Healthy professional dancers	Hand-to-foot BIA (probably, exact device not specified)	Hydrostatic Weighing (densitometry)	FFM = 0.247×Weight + 0.214×Height^2/Resistance + 0.191×Height − 14.96	Weight (kg), Height (cm), Resistance (Ohm); 50 kHz	0.83 / 1.45	Hand-to-foot (assumed)
FFM (Left Arm)	Choi et al. [15]	75 (75/0)	43.6 ± 12 years	South Korea (unilateral amputees); Unilateral amputees, male	InBody S10 (50 kHz)	DXA	-3.759 + 0.204 × (Height^2/Resistance_LA) + 0.410 × Reactance_LA + 0.019 × Height − 0.007 × Age	Height in cm, Resistance and Reactance in ohms at 50 kHz, Age in years	0.898 / 0.286 kg	hand-to-foot (segmental)
FFM (Left Leg)	Choi et al. [15]	75 (75/0)	43.6 ± 12 years	South Korea (unilateral amputees); Unilateral amputees, male	InBody S10 (50 kHz)	DXA	-4.089 + 0.162 × Reactance_LL + 0.143 × (Height^2/Resistance_LL) + 0.039 × Weight + 0.006 × Resistance_LL	Height in cm, Resistance and Reactance in ohms at 50 kHz, Weight in kg	0.908 / 0.909 kg	hand-to-foot (segmental)
FFM (Right Arm)	Choi et al. [15]	75 (75/0)	43.6 ± 12 years	South Korea (unilateral amputees); Unilateral amputees, male	InBody S10 (50 kHz)	DXA	-1.370 + 0.212 × (Height^2/Resistance_RA) + 0.054 × Reactance_RA	Height in cm, Resistance and Reactance in ohms at 50 kHz	0.736 / 0.402 kg	hand-to-foot (segmental)
FFM (Right Leg)	Choi et al. [15]	75 (75/0)	43.6 ± 12 years	South Korea (unilateral amputees); Unilateral amputees, male	InBody S10 (50 kHz)	DXA	-3.715 + 0.009 × Resistance_RL + 0.152 × (Height^2/Resistance_RL) + 0.139 × Reactance_RL + 0.031 × Weight	Height in cm, Resistance and Reactance in ohms at 50 kHz, Weight in kg	0.937 / 0.739 kg	hand-to-foot (segmental)
FFM (Trunk)	Choi et al. [15]	75 (75/0)	43.6 ± 12 years	South Korea (unilateral amputees); Unilateral amputees, male	InBody S10 (50 kHz)	DXA	-12.061 + 0.046 × (Height^2/Resistance_TR) + 0.073 × Weight + 0.212 × Height − 0.419 × Resistance_TR + 0.041 × Age	Height in cm, Resistance in ohms at 50 kHz, Weight in kg, Age in years	0.775 / 1.510 kg	hand-to-foot (segmental)
TBW	Aglago et al. [69]	250 (56/194)	18–64 years	Moroccan, North-African adults; Healthy adults	Quadscan 4000 (Bodystat, Isle of Man, UK)	Deuterium dilution (^2H_2O)	TBW (l) = 5.68 + 0.267 × (Height^2/Resistance) + 4.42 × Sex + 0.225 × Weight - 0.052 × Age	Height in cm, Resistance in ohms, Weight in kg, Age in years, Sex (male=1, female=0), measured at 50 kHz	0.92 / 1.75 l	Tetrapolar (wrist-to-ankle, supine position)
TBW	Caicedo-Eraso et al. [49]	60 (0/60)	18–24 years	Colombia (adult women); Healthy young adult women	Hydra 4200 (Xitron Technologies)	Deuterium dilution (D_2O)	TBW (L) = 0.712 × (Height^2/Resistance50) + 0.092 × Weight + 0.021 × Resistance50 - 17.216	Height in cm, Resistance at 50 kHz in ohms, Weight in kg, measured at 50 kHz	0.84 / 1.34 L	Tetrapolar (wrist-to-ankle)

TBW	Chumlea et al. [3]	15903 (7716/ 8187)	12–80 years	USA (NH White, NH Black, Mexican-American); General US population sample	Valhalla 1990B (converted to RJL equivalent)	Isotope dilution (^{2}H)	TBW (kg) = 1.203 + 0.176·weight + 0.449·(height^2 / resistance)	Height^2 in cm^2, weight in kg, resistance at 50 kHz	0.84 / 3.81 kg	Hand-to-foot (tetrapolar)
TBW	Chumlea et al. [3]	8187 (0/8187)	12–80 years	USA (NH White, NH Black, Mexican-American); General US population sample	Valhalla 1990B (converted to RJL equivalent)	Isotope dilution (^{2}H)	TBW (kg) = 3.747 + 0.113·weight + 0.450·(height^2 / resistance)	Height^2 in cm^2, weight in kg, resistance at 50 kHz	0.79 / 2.61 kg	Hand-to-foot (tetrapolar)
TBW	Devakumar et al. [76]	102 (50/52)	7–9 years	Nepal (rural children); Healthy children	Tanita BC-418 (segmental, 50 kHz)	Deuterium dilution ($^{2}H_{2}O$)	TBW (kg) = 0.7146 + 1.5959 × (Height^2/Impedance)	Height in cm, Impedance in ohms at 50 kHz	0.92 / 0.781 kg	Foot-to-foot (leg-to-leg, 180° arms)
TBW	Devakumar et al. [76]	102 (50/52)	7–9 years	Nepal (rural children); Healthy children	Tanita BC-418 (segmental, 50 kHz)	Deuterium dilution ($^{2}H_{2}O$)	TBW (kg) = 0.6667 + 1.6324 × (Height^2/Impedance)	Height in cm, Impedance in ohms at 50 kHz	0.92 / 0.775 kg	Foot-to-foot (leg-to-leg, 90° arms)
TBW	Diouf et al. [71]	151 (74/77)	8–11 years	Senegalese children; Healthy, prepubertal children	Xitron 4000B (Xitron Technologies, USA)	Deuterium dilution method (DDM)	TBW (kg) = 0.376 × (Height^2/Z_{50}) - 0.470 × Sex + 0.076 × Weight + 0.065 × Height - 2.28	Height in cm, Weight in kg, Resistance Z_{50} $in\ ohms$, Sex (male=1, female=2), measured at 50 kHz	0.89 / 0.89 kg	Tetrapolar (left side: wrist, hand, foot, ankle)
TBW	Divala et al. [13]	186 (79/107)	10–18 years	Malawi; Healthy adolescents	Bodystat 1500 MDD (Bodystat Ltd)	Deuterium dilution (DDM)	TBW (kg) = -2.152 + 0.328 × (Height^2/Resistance) + 0.910 × Sex + 0.307 × Age + 0.249 × Weight + 0.015 × Reactance	Height in cm, Resistance in ohms, Weight in kg, Age in years, Sex (male=1, female=0), Reactance in ohms, measured at 50 kHz	0.922 / 1.47 kg	Tetrapolar (wrist-to-ankle)
TBW	El Harchaoui et al. [19]	247 (122/ 125)	8–11 years	Morocco; Healthy school-aged children	Bodystat QuadScan 4000 (50 kHz)	Deuterium dilution	0.296 + 0.292 × (Height^2/Resistance) + 0.221 × Weight + 0.291 × Age + 0.824 × Sex (male=1, female=0)	Height in cm, Resistance in ohms at 50 kHz, Weight in kg, Age in years, Sex coded (male=1, female=0)	0.911 / 1.514 kg	hand-to-foot
TBW	Essa'a et al. [47]	102 (47/55)	24–60 months	Cameroon; Healthy preschool children	Bodystat QuadScan 4000 (50 kHz)	Deuterium dilution	-6.206 + 0.0037 × (Height^2/Resistance) - 0.0410 × Age (months) + 0.2652 × Weight (kg) + 0.1214 × Height (cm)	Height in cm, Resistance in ohms at 50 kHz, Weight in kg, Age in months	0.68 / 1.4 kg	hand-to-foot
TBW	Haroun et al. [75]	382 (181/201)	11–15 years	UK (White, Black, Asian adolescents); Healthy adolescents	TANITA TBF-300 (leg-to-leg, 50 kHz)	Deuterium dilution ($^{2}H_{2}O$)	TBW (L) = -1.822 + 0.665 × (Height^2/Resistance) + 1.166 × Black + 1.288 × Asian	Height in cm, Resistance in ohms at 50 kHz; 'Black' and 'Asian' as dummy variables (1/0)	0.9 / 2.85 L	Foot-to-foot
TBW	Haroun et al. [75]	382 (181/ 201)	11–15 years	UK (White, Black, Asian adolescents); Healthy adolescents	TANITA TBF-300 (leg-to-leg, 50 kHz)	Deuterium dilution ($^{2}H_{2}O$)	TBW (L) = 0.125 + 0.647 × (Height^2/Resistance) + 2.187 × Black + 1.465 × Asian	Height in cm, Resistance in ohms at 50 kHz; 'Black' and 'Asian' as dummy variables (1/0)	0.85 / 2.36 L	Foot-to-foot

TBW	Hastuti et al. [70]	292 (292/0)	18–65 years	Indonesian (Javanese ethnicity); Healthy adult men	Imp DF50 (ImpediMed Ltd., Australia)	Deuterium dilution (^2H_2O)	TBW (kg) = 0.167 + 0.213 × Body Weight + 0.327 × Resistance Index + 0.055 × Reactance	Body Weight in kg, Resistance Index (Height^2/Resistance) with Height in cm and Resistance in ohms, Reactance in ohms, measured at 50 kHz	0.836 / 1.8 kg	Tetrapolar (wrist-to-ankle, supine position)
TBW	Jayawardena et al. [44]	170 (56/114)	19–69 years	Sri Lanka; Healthy adults	InBody 230 (multi-frequency analyzer)	Deuterium dilution	-22.037 + 0.433 × (Height^2/Resistance) + 2.340 × Gender (male=1, female=0) + 0.095 × WC + 0.110 × HC + 0.130 × Height	Height, WC, HC in cm; Resistance in ohms; Gender binary coded (male=1, female=0)	0.732 / 5.06 kg	hand-to-foot
TBW	Khatun et al. [74]	1500 (1500/0)	Wide (NHANES sample)	USA; General population	50 kHz BIA (device unspecified)	Deuterium dilution (^{2}H□O) + DXA	TBW (L) = -10.989 + 0.013 × Age + 0.472 × BMI + 0.004 × Z_{50} + 0.634 × (Height^2/Z_{50})	Height in cm, BMI in kg/m^2, Z_{50} impedance in ohms at 50 kHz, Age in years	0.998 / 0.82 L	Unspecified
TBW	Khatun et al. [74]	1500 (0/1500)	Wide (NHANES sample)	USA; General population	50 kHz BIA (device unspecified)	Deuterium dilution (^{2}H□O) + DXA	TBW (L) = -9.028 - 0.001 × Age + 0.357 × BMI + 0.004 × Z_{50} + 0.625 × (Height^2/Z_{50})	Height in cm, BMI in kg/m^2, Z_{50} impedance in ohms at 50 kHz, Age in years	0.997 / 0.69 L	Unspecified
TBW	Khatun et al. [73]	1397 (1397/0)	nan	US citizens (NHANES sample); Mixed healthy population	HYDRA Bio-Impedance Spectrum Analyzer (Model 4200, Xitron Technologies, USA)	Deuterium dilution (D□O)	TBW (L) = -11.25 + 0.011 × Age + 0.46 × BMI + 0.005 × Z□□□ + 0.60 × (Height^2/Z□□□)	Height in cm, BMI in kg/m^2, Z□□□ impedance in ohms at 100 kHz, Age in years, measured at 100 kHz	0.998 / 0.76 L	Tetrapolar (wrist-to-ankle, supine)
TBW	Khatun MF et al. [73]	1420 (0/1420)	nan	US citizens (NHANES sample); Mixed healthy population	HYDRA Bio-Impedance Spectrum Analyzer (Model 4200, Xitron Technologies, USA)	Deuterium dilution (D□O)	TBW (L) = -10.56 - 0.002 × Age + 0.36 × BMI + 0.006 × Z□□□ + 0.60 × (Height^2/Z□□□)	Height in cm, BMI in kg/m^2, Z□□□ impedance in ohms at 100 kHz, Age in years, measured at 100 kHz	0.997 / 0.58 L	Tetrapolar (wrist-to-ankle, supine)
TBW	Kourkoumelis et al. [18]	266 (143/123)	7.3–14.5 years	Europe (Bosnia and Herzegovina, Latvia, Montenegro, North Macedonia, Portugal); Healthy children and adolescents	Impedimed DF50 and BodyStat 1500MDD (50 kHz)	Deuterium dilution	TBW = 0.44 × (Height^2/Resistance) + 0.12 × Weight + 0.33 × Sex (male=1, female=0) + 1.5	Height in cm, Resistance in ohms at 50 kHz, Weight in kg, Sex binary coded	0.87 / 1.81 kg	hand-to-foot
TBW	Leman et al. [66]	39 (17/22)	5–18 years	Yoruba children, south-western Nigeria; Healthy children	BIA 01Q (RJL Systems)	Deuterium dilution (^{2}H□O)	TBW (kg) = 1.67 + 0.35 × (Height^2/Resistance) + 0.24 × Weight - 0.74 × Gender	Height in cm, Resistance in ohms, Weight in kg, Gender (male=0, female=1), measured at 50 kHz	0.987 / 0.7 kg	Tetrapolar (wrist-to-ankle, standing posture, corrected resistance)

TBW	Leman et al. [66]	92 (42/50)	5–65 years	Yoruba population, south-western Nigeria; Healthy individuals	BIA 01Q (RJL Systems)	Deuterium dilution (^{2}H□O)	TBW (kg) = 1.93 + 0.47 × (Height^2/Resistance) + 0.13 × Weight - 1.20 × Gender	Height in cm, Resistance in ohms, Weight in kg, Gender (male=0, female=1), measured at 50 kHz	0.97 / 1.7 kg	Tetrapolar (wrist-to-ankle, standing posture, corrected resistance)
TBW	Lewis et al. [17]	50 (27/23)	12–59 months	Ugandan; Stunted children (HAZ < -2), non-SAM, no medical complications	Bodystat 500	Deuterium dilution (^{2}H)	TBW (kg) = 3.050 + 0.333·sex + 0.049·age + 0.312·(H^2/Z_{50}) + 0.167·HAZ	Height^2 in cm^2, impedance at 50 kHz, age in months, sex (male=1, female=0), HAZ (Z-score)	0.942 / 0.311 kg	Hand-to-foot (tetrapolar, right side)
TBW	Liu et al. [68]	630 (328/ 302)	8–10 years	Chinese, Lebanese, Malaysian, Filipino, Thai; Healthy, prepubertal (Tanner stage 1) children	Imp DF50 (ImpediMed Limited, Australia)	Deuterium dilution (^{2}H□O)	TBW (kg) = 0.231 × (Height^2/Resistance) + 0.066 × Height + 0.188 × Weight + 0.128 × Age + 0.500 × Sex - 0.316 × Ethnicity (Thai) - 4.574	Height in cm, Resistance in ohms, Weight in kg, Age in years, Sex (male=1, female=0), Ethnicity dummy (Thai=1, others=0), measured at 50 kHz	0.88 / 1.3 kg	Tetrapolar (wrist-to-ankle, right side)
TBW	Matias et al. [2]	208 (138/70)	18–30 (mean ~21)	National-level Portuguese athletes; Healthy, national-level athletes	BIA-101 RJL/Akern Systems (50 kHz)	Deuterium dilution	TBW = 0.286 + 0.195×Stature^2/Resistance + 0.385×Weight + 5.086×Sex (Sex: Male=1, Female=0)	Weight (kg), Stature (cm), Resistance (Ohm); 50 kHz	0.93 / 2.42	Hand-to-foot
TBW	Maw et al. [12]	102 (57/45)	4–8 years	Myanmar (rural children); Healthy rural children	BIM 4, ImpediMed Ltd., Brisbane (50 kHz)	Deuterium dilution (^{2}H□O)	TBW (kg) = 0.4597 × Weight (kg) + 0.1564 × (Height^2/Resistance) + 0.6075	Weight in kg, Height in cm, Resistance in ohms at 50 kHz	0.891 / Not directly stated; LOA ±1.0 kg	Tetrapolar (hand-to-foot)
TBW	Shaikh et al. [64]	82 (0/82)	Postpartum, ~20 years	Bangladesh; Postpartum rural women	RJL Systems (Single-frequency BIA)	Deuterium dilution (^{2}H□O)	TBW (kg) = 4.297 + 0.190 × Weight + 0.349 × (Height^2/Resistance)	Height in cm, Resistance in ohms, Weight in kg, measured at 50 kHz	0.78 / 1.30 kg	Tetrapolar (hand-to-foot)
TBW	Shaikh et al. [64]	147 (0/147)	Postpartum, ~20 years	Bangladesh; Postpartum rural women	Quadscan 2000 (BodyStat Ltd, Multi-frequency BIA)	Deuterium dilution (^{2}H□O)	TBW (kg) = 4.573 + 0.177 × Weight + 0.351 × (Height^2/Z50)	Height in cm, Z50 (impedance) in ohms, Weight in kg, measured at 50 kHz	0.76 / 1.32 kg	Tetrapolar (hand-to-foot)
TBW	Sun et al. [5]	712 (712/0)	12–94 years	USA (white and black); Healthy adults, broad population sample	Valhalla BIA device, RJL equivalent	Multicomponent model	TBW (L) = 1.20 + 0.45·(stature^2 / resistance) + 0.18·weight	Height^2 in cm^2, resistance in ohms, weight in kg, single frequency 50 kHz	0.84 / 3.8 L	Hand-to-foot (tetrapolar)
TBW	Sun et al. [5]	1089 (0/1089)	12–94 years	USA (white and black); Healthy adults, broad population sample	Valhalla BIA device, RJL equivalent	Multicomponent model	TBW (L) = 3.75 + 0.45·(stature^2 / resistance) + 0.11·weight	Height^2 in cm^2, resistance in ohms, weight in kg, single frequency 50 kHz	0.79 / 2.6 L	Hand-to-foot (tetrapolar)

TBW	Waidyatilaka et al. [65]	80 (0/80)	30–45 years	Urban Sri Lankan women; Healthy women	SFB7 ImpediMed analyzer (ImpediMed Ltd, Australia)	Deuterium dilution (^{2}H□O)	TBW (kg) = 3.443 + 0.342 × (Height^2/Impedance) + 0.176 × Weight	Height in cm, Impedance in ohms, Weight in kg, measured at 50 kHz	0.876 / 1.34 kg	Tetrapolar (wrist-to-ankle)
TBW	Wickramasinghe et al. [63]	282 (158/ 124)	5–15 years	Sri Lanka; Healthy children	Bodystat (Bodystat Ltd, Isle of Man)	Deuterium dilution (D□O)	TBW (l) = 0.41 × (Height^2/Impedance) + 0.17 × Weight + 1.1 × Sex + 0.44	Height in cm, Impedance in ohms, Weight in kg, Sex (male=1, female=0), measured at 50 kHz	0.863 / 2.1 l	Tetrapolar (hand-to-foot)
